# Geographical distribution and species variation of gut microbiota in small rodents from the agro‐pastoral transition ecotone in northern China

**DOI:** 10.1002/ece3.11084

**Published:** 2024-03-11

**Authors:** Yongzhen Wu, Taoxiu Zhou, Chen Gu, Baofa Yin, Shengmei Yang, Yunzeng Zhang, Ruiyong Wu, Wanhong Wei

**Affiliations:** ^1^ College of Bioscience and Biotechnology Yangzhou University Yangzhou Jiangsu China

**Keywords:** 16S rRNA gene sequencing, distance isolation, gut microbiota, rodent

## Abstract

The gut microbiota of rodents is essential for survival and adaptation and is susceptible to various factors, ranging from environmental conditions to genetic predispositions. Nevertheless, few comparative studies have considered the contribution of species identity and geographic spatial distance to variations in the gut microbiota. In this study, a random sampling survey encompassing four rodent species (*Apodemus agrarius*, *Cricetulus barabensis*, *Tscherskia triton* and *Rattus norvegicus*) was conducted at five sites in northern China's farming–pastoral ecotone. Through a cross‐factorial comparison, we aimed to discern whether belonging to the same species or sharing the same capture site predominantly influences the composition of gut microbiota. Notably, the observed variations in microbiome composition among these four rodent species match the host phylogeny at the family level but not at the species level. The gut microbiota of these four rodent species exhibited typical mammalian characteristics, predominantly characterized by the Firmicutes and Bacteroidetes phyla. As the geographic distance between populations increased, the number of shared microbial taxa among conspecific populations decreased. We observed that within a relatively small geographical range, even different species exhibited convergent α‐diversity due to their inhabitation within the same environmental microbial pool. In contrast, the composition and structure of the intestinal microbiota in the allopatric populations of *A. agrarius* demonstrated marked differences, similar to those of *C. barabensis*. Additionally, geographical environmental elements exhibited significant correlations with diversity indices. Conversely, host‐related factors had minimal influence on microbial abundance. Our findings indicated that the similarity of the microbial compositions was not determined primarily by the host species, and the location of the sampling explained a greater amount of variation in the microbial composition, indicating that the local environment played a crucial role in shaping the microbial composition.

## Graphical abstract

This study explored the gut microbiota of four rodent species in northern China and found that microbial composition was more influenced by geographic location than host species, highlighting the pivotal role of the local environment in shaping gut microbiota.

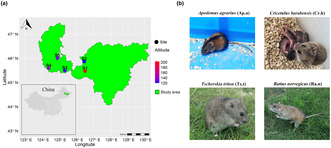

## 1 INTRODUCTION

Microbes are considered an integral animal phenotype component (Holmes & Nicholson, 2005), exerting an influence on the fitness and consequently on the ecologically significant traits of their hosts (Greene et al., 2020; Groussin et al., 2017; McFall‐Ngai et al., 2013). Despite the increasing number of studies dedicated to unveiling the mysterious factors that determine the structure of the host gut microbiota, many gaps persist, especially in various animal hosts within natural systems.

In recent decades, studies based on a combination of bioinformatic analysis and high‐throughput sequencing technology have shown that many factors, such as host genetics, diet, season, age and lifestyle, can strongly affect the gut microbiota compositions (Adriansjach et al., 2020; Benson et al., 2010; Claesson et al., 2012; Jiang et al., 2021; Kavaliers et al., 2021; Reese et al., 2021; Spor et al., 2011). However, there is still debate about the precise nature of the predominant factors, with some researchers focusing on the role of genetics, while others highlighting environmental influences. For instance, research conducted in laboratory settings has revealed a significant correlation between the genetic makeup of mice and the composition of their gut microbiota, whereas co‐housing has a limited impact on microbiota composition, emphasizing the role of host genetics (Campbell et al., 2012). Additionally, studies in natural environments have revealed that species‐specific components of microbial composition stem from the shared diversification of both the host and microbes. A study that compared the gut microbiota of small mammals (especially mice, voles and shrews) in various habitats ranging from 2 to 23 km found that despite factors such as diet and location in the environment that influence the host's microbiota to some extent, species identity was the strongest microbial composition predictor (Knowles et al., 2019). This conclusion is consistent with previous studies on wood mice (Weinstein et al., 2021) and American pikas (Kohl, Dearing, & Bordenstein, 2018a; Kohl, Varner, et al., 2018b).

Moreover, some studies have emphasized the decisive role of environmental factors. Dietary variations can have a profound influence on gut microbiota. A study using genetically defined mice revealed that dietary intervention, transitioning mice to a high‐glucose, high‐fat diet, could rapidly reshape the abundance of the gut microbial community into a novel and stable composition, independent of hereditary distinctions between individual mice (Carmody et al., 2015). This finding is consistent with that of a previous study involving humans (David et al., 2014). Furthermore, insights from nonhuman primates suggest that the gut microbiota of baboons is primarily influenced by soil geological history and exchangeable sodium content, rather than host genetic factors (Grieneisen et al., 2019). Similarly, research on humans and four species of hominoids revealed a weak correspondence between the phylogeny of host systems based on mitochondrial DNA (mtDNA) and the composition of their faecal microbiota. This underscores the significance of host environment (Ochman et al., 2010).

Although research involving wild and laboratory animals provides substantial evidence regarding environmental and genetic influences, it is essential to consider several factors that can introduce bias into research conclusions. For example, some studies that emphasize genetic dominance in the variation of host‐associated microbial communities face challenges because they are restricted to laboratory settings or relatively small geographic scales (Wang et al., 2019). These limitations hinder the detection of factors that influence the microbiota of species with strong intestinal dissemination capabilities (Pascoe et al., 2017). However, studies that emphasize environmental factors often focus on a limited number of species or individual factors and fail to comprehensively address the interactions among these factors. Given the lack of correlation between laboratory model mouse studies and ecological environments, conducting research in natural habitats with greater geographic distances can mitigate the confounding effects of intraspecific gene flow, enabling the examination of the major factors contributing to variation in host gut microbiota (Lin et al., 2020; Linnenbrink et al., 2013). This approach can significantly improve our understanding of the factors that shape microbial communities and their dynamics.

The ecological environment of the agro‐pastoral transition ecotone has become sensitive, fragile and unstable due to ongoing transitional geographical climate changes and human disturbances (Wang et al., 2015). Consequently, it has evolved into a typical ecologically vulnerable belt. These environmental characteristics inherently provide conducive conditions for the survival and reproduction of rodent species. *A. agrarius* is a member of the Muridae family within the Apodemus genus. Distinguished by its varied diet, it predominantly feeds on plant‐based foods (Balciauskas et al., 2019, 2021), showcasing its adaptability to diverse environmental conditions. *R. norvegicus*, a species within the Muridae family's Rattus genus, exhibits near‐global ubiquity, excluding polar ice caps (Puckett et al., 2016). Adult individuals of this species typically measure 15–25 cm in length and weigh between 220 and 280 g. *R. norvegicus* maintains an omnivorous diet encompassing virtually all food items consumed by humans. It particularly favours high‐moisture fruits and vegetables, as well as staples such as rice and wheat (Guiry & Buckley, 2018). *C. barabensis* and *T. triton*, both belonging to the Cricetulus genus of the Cricetinae subfamily, exhibit a preference for consuming plant seeds (Yu et al., 2020; Zhao et al., 2022). The structural composition and dynamics of these species' communities serve as direct indicators of the ecological conditions in the ecotone, and their notable fecundity and broad geographical distribution underscore their sensitivity to environmental fluctuations.

In this study, we employed 16S rRNA gene‐sequencing technology in conjunction with bioinformatic analysis to characterize the microbial communities of these organisms at both the individual and population levels. Furthermore, we used principal coordinate analysis (PCoA), permutational multivariate analysis of variance (PERMANOVA), Spearman correlation analysis and other methodologies to quantify the contributions of host‐related and geographic factors to the variation in gut microbiota communities. This study aimed to demonstrate that as the spatial distance between populations increases, the congruence between the phylogenetic signals of host gut microbiota and the evolutionary overlap among hosts decreases, while community disparities escalate per the geographic distance between populations. We hope that this study will contribute to a more comprehensive understanding of the interactions between host genetics and environmental factors, as well as their significance in shaping gut microbiota composition.

## 2 MATERIAL AND METHOD

### 2.1 Sample and field data collection

The study was conducted in the agro‐pastoral transition ecotone in Northern China, known for its diverse agricultural landscapes. We randomly set five survey sites in the farming–pastoral ecotone with a distance of 30–50 km to verify the distance attenuation hypothesis of host gut microbiota similarity. The specific locations were as follows: Dongsheng Village, Hongqi Manchu Township, Nangang District, Harbin City, Heilongjiang Province (S1: 45°57 N, 126°48 E, elevation 182 m); Houchaitun, Wanbao Town, Songbei District, Harbin City, Heilongjiang Province (S2: 45°87 N, 126°37 E, elevation 120 m); Minzhu Village, Erzhan Town, Zhaoyuan County, Daqing City, Heilongjiang Province (S3: 45°50 N, 125°31 E, elevation 124 m); Jianmin Village, Xinzhan Town, Zhaoyuan County, Daqing City, Heilongjiang Province (S4: 45°59 N, 124°37 E, elevation 139 m); and Xiangfang Village, Datong Town, Datong District, Daqing City, Heilongjiang Province (S5: 46°01 N, 124°88 E, elevation 132 m). Within each sampling site, four transects were established, and on each transect, 75 small collapsible aluminium Sherman traps (2 × 2.5 × 6.5 inches) baited with peanuts were deployed. To mitigate the impact of habitat types, the sampling transects were strategically positioned in corn‐dominated agricultural fields at each site. The traps were set continuously for 2 consecutive days, using the dusk‐to‐dawn method. Captured animals were immediately euthanized using ether. Then, individual metrics, including gender, weight, body length, tail length and skull length, were recorded for each sample (Table S1). Geo‐coordinates of sampling sites were documented, and climate data, including altitude, annual average temperature and annual precipitation, were obtained from global climate databases based on these coordinates (Table S2). All animals were stored in sterile bags at −20°C until dissection.

**FIGURE 1:**
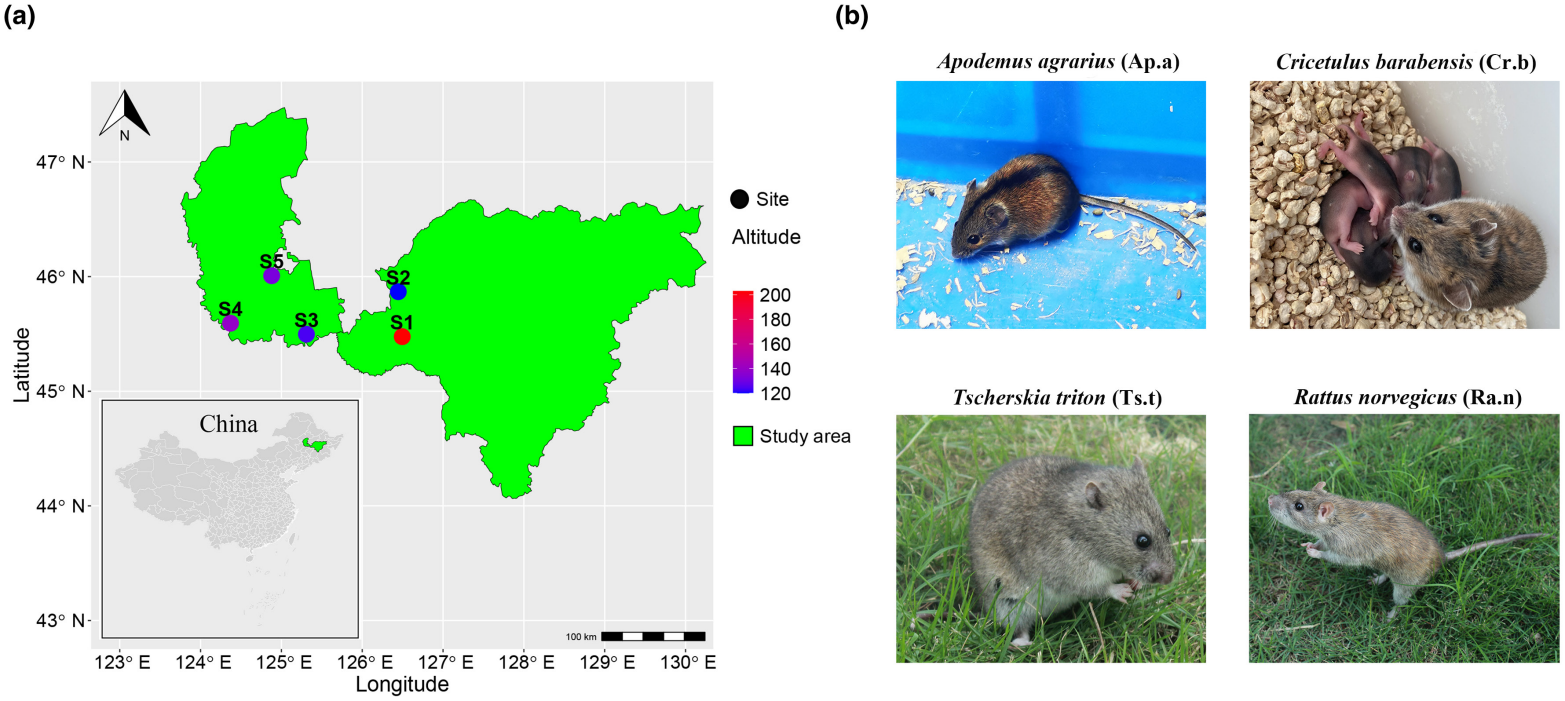
The geographical location information of the sampling sites (a) and photos of species studied (b). (S1: Dongsheng Village, DS; S2: Wanbao Town, WB; S3: Erzhan Town, EZ; S4: Xinzhan Town, XZ; S5: Datong Town, DT; Ap.a: *Apodemus agrarius*, Cr.b: *Cricetulus barabensis*, Ts.t: *Tscherskia triton*, Ra.n: *Rattus norvegicus*).

In total, 110 rodents of four species (*R. norvegicus*, *A. agrarius*, *C. barabensis* and *T. triton*) were collected between 24 July and 17 August 2020 (Figure 1 and Table S3). We thoroughly cleaned the dissection tools with 75% ethanol before performing sample dissection. Approximately 10 mg of content from the distal caecum was extracted as a microbial sample. To prevent RNA degradation within the microbial samples, the caecum contents were placed in 4 mL of RNALater. At the end of the fieldwork, the samples were transported while frozen in the laboratory and stored at −80°C before DNA extraction.

All experimental procedures adhered to ethical guidelines, ensuring minimal impact on the subjects and standardized laboratory protocols were followed to maintain data reliability and repeatability.

### 2.2 DNA extraction and sequencing

The whole‐genome DNA of the samples was extracted using the CTAB method, and the DNA concentration and purity of the 1% DNA in the agarose gels were checked. According to the concentration, DNA was diluted to 1 ng/μL using sterile water. Using primers 515F (5′‐barcode‐GTGCCAGCMGCCGCGGTAA‐3′) and 806R (5′‐GGACTACHVGGGTWTCTAAT‐3′) to amplify the 16S rRNA genes in the V4 region. All PCR reactions were performed using 15 μL of Phusion® High‐Fidelity PCR Master Mix (New England Biolabs), 2 μM of forward and reverse primers and approximately 10 ng template DNA. The thermal cycle ageing from 98°C for 1 min. Then, denature at 98°C for 30 cycles for 10 s, annealing at 50°C for 30 s and 72°C tensile strength for 30 s. Finally, they were kept at 72°C for 5 min. Next, we mixed an equal amount of 1× loaded buffer (containing SYB green) with the PCR products and examined them by electrophoresis on a 2% agarose gel. PCR products were mixed in equidensity ratios, and the mixture was purified using the Qiagen Gel Extraction Kit (Qiagen, Germany). Sequencing libraries were generated using the TruSeq® DNA PCR‐Free Sample Preparation Kit (Illumina, USA) following the manufacturer's recommendations and index codes were added. Library quality was assessed using a Qubit@ 2.0 Fluorometer (Thermo Scientific) and an Agilent Bioanalyzer 2100 system. Finally, the library was sequenced on an Illumina NovaSeq platform and 250 bp paired‐end target reads were generated.

### 2.3 16S rRNA bioinformatics

Quality control of the demultiplexed paired‐end sequence reads was performed according to QIIME2 (Caporaso et al., 2010). First, paired‐end reads were assigned to samples based on their unique barcodes and truncated by cutting off the barcodes and primer sequences. The reads were merged using FLASH (version 1.2.11, http://ccb.jhu.edu/software/FLASH/) (Magoc & Salzberg, 2011), and the spliced sequences were called raw tags. Quality filtering of the raw tags was performed using the Fastp software (version 0.20.0) to obtain clean high‐quality tags. Clean tags were compared with a reference database (Silva database https://www.arbsilva.de/) using Vsearch (version 2.15.0) to detect chimeric sequences, which were then removed to obtain effective tags (Haas et al., 2011). Subsequently, effective tags were denoised using the DADA2 module in the QIIME2 software to obtain initial sequence variants of amphibians (ASVs), and ASVs with an abundance of under 5 were filtered out (Marizzoni et al., 2020). To study the phylogenetic relationship of each ASV and the differences in the dominant species among different samples, multiple‐sequence alignments were performed using the QIIME2 software. The absolute abundance of the ASVs was normalized using a standard sequence number corresponding to the sample with the lowest number of sequences. Subsequent analyses of α‐ and β‐diversities were performed based on the output normalized data.

### 2.4 Statistical analyses

All analyses were conducted using R version 4.3.1. To assess species richness and diversity within and between samples or groups, the ‘vegan’ R package was employed to calculate the Chao1 and Shannon α‐diversity indices, and non‐parametric Mann–Whitney *U* test and Krystal–Wallis test were performed to assess the differences in diversity indexes between two groups and among multiple groups respectively. To assess differences in the microbial community composition between samples, we conducted permutational multivariate analysis of variance (PERMANOVA) using the Bray–Curtis dissimilarity. Adonis function in the ‘vegan’ package was employed for PERMANOVA analysis. Changes in bacterial composition within samples were observed using principal coordinates analysis (PCoA). PCoA was conducted using the ‘vegan’ R package. To examine the relative contributions of species and capture sites in predicting the microbial community composition of each host genus, we obtained evolutionary distances for the four species from the TimeTree website (https://timetree.org/). Subsequently, we calculated average microbial community composition for all samples of each species and performed hierarchical clustering based on average linkage for the Bray–Curtis dissimilarity. This generated a dendrogram representing the microbial community composition. To investigate whether there are significant differences in the abundance of specific taxa between different groups of conspecific populations as well as heterospecific populations, linear discriminant analysis (LDA) effect size (LEfSe) (https://huttenhower.sph.harvard.edu/lefse/) was performed on a normalized relative abundance matrix. Furthermore, we utilized Spearman correlation analysis to evaluate the correlation between the Bray–Curtis similarity matrix of ASV and the geographical distance matrix and examined the correlations between environmental factors, α‐diversity indices and microbial community composition.

## 3 RESULTS

### 3.1 Identification of bacterial ASVs

16S rRNA amplicon sequencing of a total of 110 rodent caecal content samples targeting the hypervariable V4 region of the 16S rRNA gene was sequenced and analysed, and 7,111,399 high‐quality sequences were obtained. Based on rarefaction curve analysis, we found Chao1 and Shannon reached high saturation (Figure S1), which indicated that almost all bacterial taxa in each sample were identified. These sequences were clustered into 6308 ASVs, representing 36 phyla, 85 classes, 183 orders, 303 families and 529 genera (Table S4).

### 3.2 Phylogenetic structure of ASVs and their differences across hosts and capture sites

**FIGURE 2:**
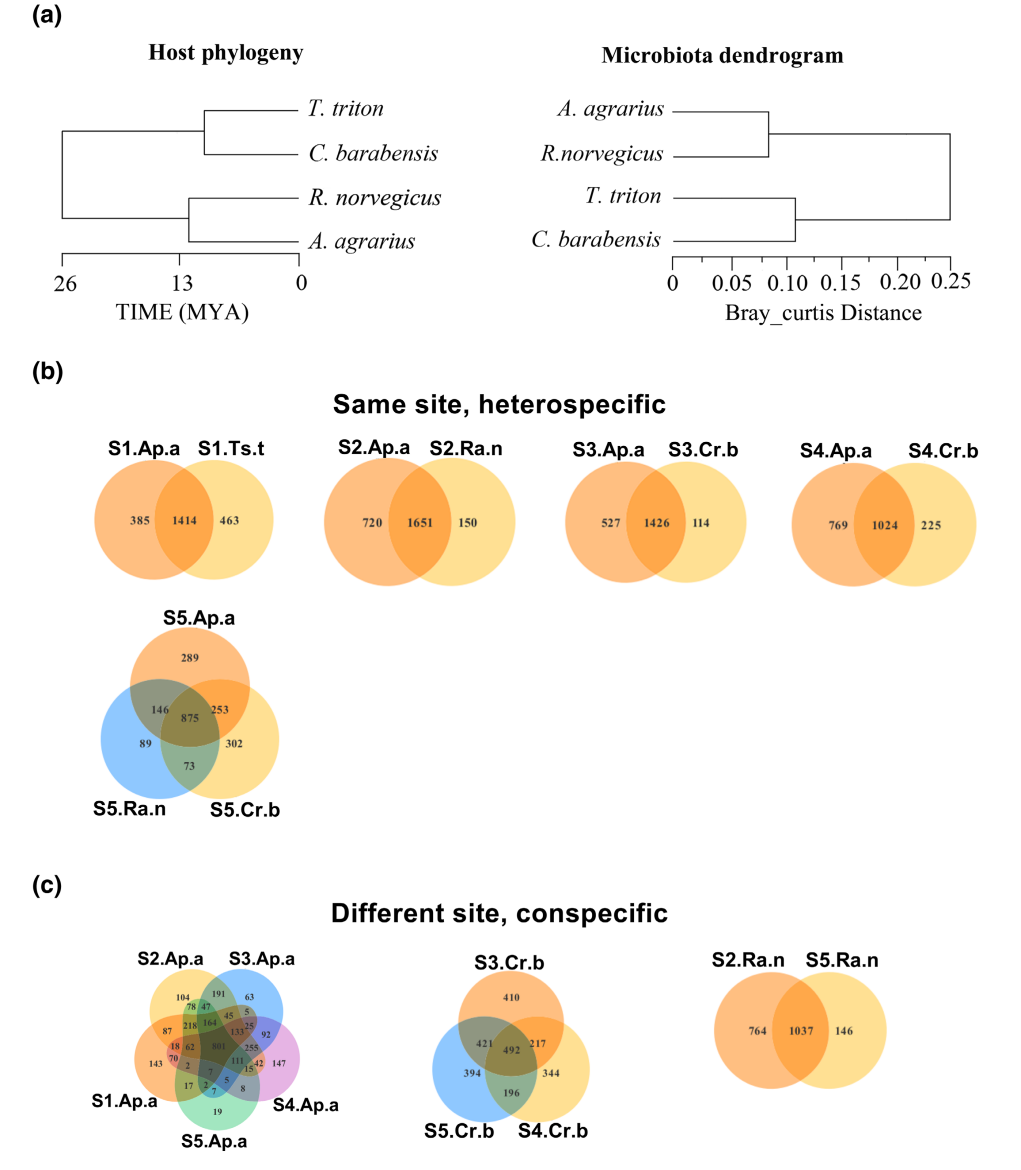
Comparison of the systematic phylogenetic structure of ASVs with dendrogram of similarity in microbiota composition in the four rodent species (a), and a comparison of their distribution among heterospecific sympatric (b) and conspecific allopatric population populations (c) (Ap.a: *Apodemus agrarius*, Cr.b: *Cricetulus barabensis*, Ts.t: *Tscherskia triton*, Ra.n: *Rattus norvegicus*).

Analysis of the full dataset showed a congruence at the family level of the host between the bacterial phylogenetic tree constructed based on Bray–Curtis distance and the host evolutionary tree, but this congruence did not match at the species level (Figure 2a).

The VEEN diagram illustrates the shared counts of ASVs across different species and populations at various sites, we could find some conservative ASVs in all comparison groups and the number of conservative bacteria was consistently higher in heterospecific populations within the same habitat than in conspecific populations in different sites (Figure 2b,c; Figure S2).

Results of PERMANOVA testing based on Bray–Curtis, weighted‐UniFrac and unweighted‐UniFrac distances matrices showed capture site significantly explained 16%–21% of the β‐diversity between samples, which was higher than that explained 4%–10% by species identity, and alongside weaker effects of host age and sex variables (Table 1). These results suggested that within host species, the microbiota is shaped more strongly by capture site than species identity, and indicated that site location and host species played different roles on the host gut microbial communities.

**TABLE 1:** PERMANOVA (adonis) tests for significance and relative contribution of environmental and phylogenetic factors to variation in Bray–Curtis and weighted‐ and unweighted‐UniFrac distance matrices constructed from rodent caecum microbiomes.

	Bray–Curtis			Unweighted UniFrac			Weighted UniFrac		
	Pseudo‐F	*R* ^2^	*p*	Pseudo‐F	*R* ^2^	*p*	Pseudo‐F	*R* ^2^	*p*
Species	2.22	.05	<.001***	1.66	.04	.009**	5.71	.10	<.001***
Sites	7.40	.21	<.001***	5.58	.16	**<.001** ***	8.53	.20	<.001***
Sex	0.85	.01	.67	1.19	.04	.11	1.36	.03	.15
Age	1.11	.03	.23	1.01	.01	.38	0.88	.01	.46

*p < .05, **p < .01 and ***p < .001.

### 3.3 Bacterial composition and abundance among populations

**FIGURE 3:**
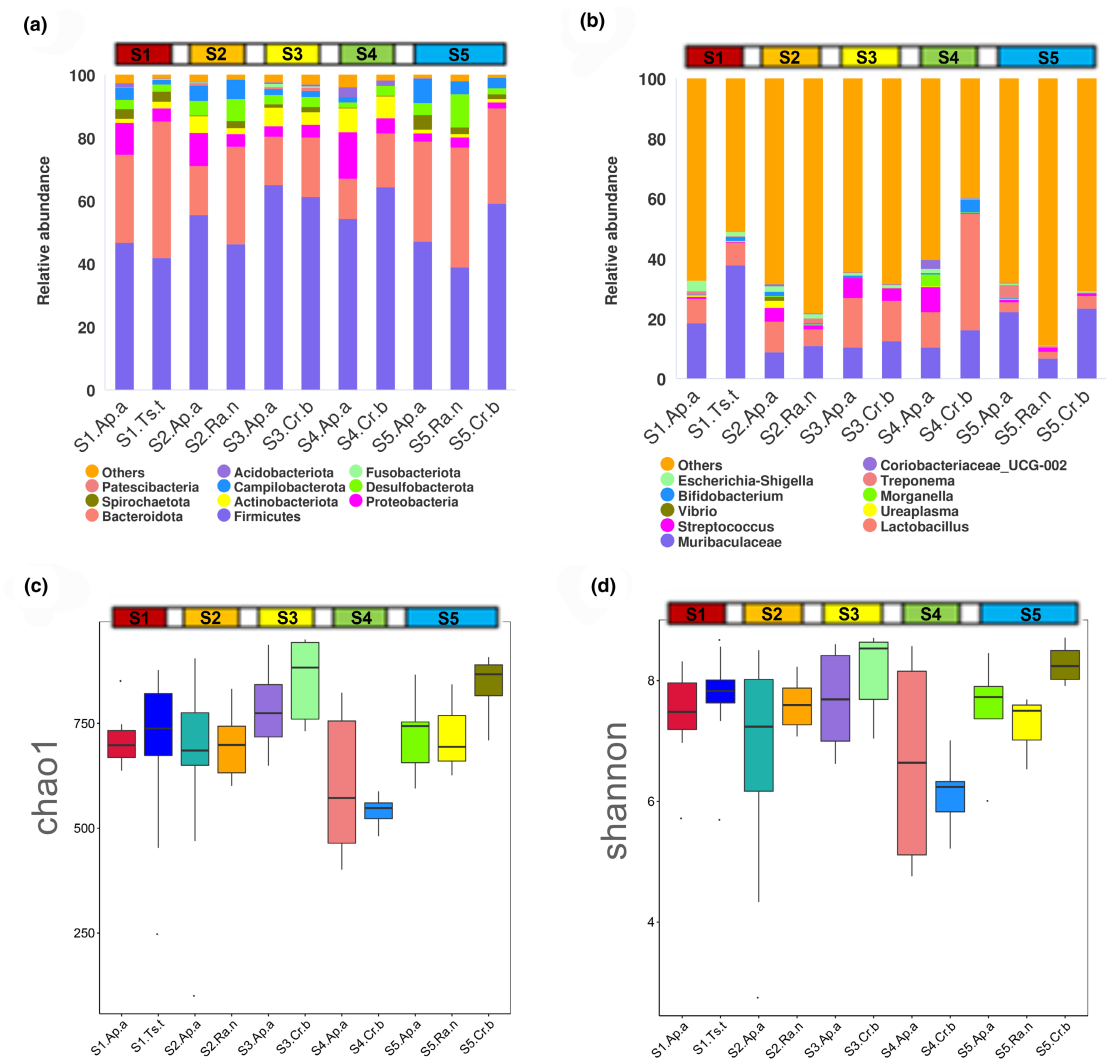
Gut microbial composition and abundance of the four rodent species from different sites. (a) Relative abundance of the top 10 species in phylum level. (b) Relative abundance of the top 10 species in genus level. (c) Variation in α‐diversity in the gut microbiota of rodents based on Chao1 index and (d) Shannon index (Ap.a: *Apodemus agrarius*, Cr.b: *Cricetulus barabensis*, Ts.t: *Tscherskia triton*, Ra.n: *Rattus norvegicus*).

The dominant phyla (mean relative abundance >10%) in all populations were Firmicutes (33.52%–57.05%, with an average relative abundance of 44.12%) and Bacteroidetes (11.53%–39.91%, average 24.32%). Rare phyla (mean relative abundance <5%) included Campilobacterota (average 3.44%), Actinobacteriota (average 3.31%), Desulfobacterota (average 1.87%), Spirochaetota (average 1.6%), Acidobacteriota (average 0.79%) and Fusobacteriota (average 0.15%) (Figure 3a).

At the genus level, the top 10 genera were *Lactobacillus* (3.8%–40.8%, average 12.32%), *Helicobacter* (0.1%–8.0%, average 3.4%), *Lachnospiraceae_NK4A136_group* (1.1%–5.4%, average 3.4%), *Streptococcus* (0.2%–6.9%, average 2.4%), *Bacteroides* (0%–14.0%, average 1.8%), *Prevotella* (0%–14.0%, average 1.7%), *Desulfovibrio* (0.8%–2.6%, average 1.5%), *Colidextribacter* (0.7%–2.3%, average 1.4%), *Roseburia* (0.2%–3.4%, average 1.4%) and *Prevotellaceae_UCG‐003* (0%–4.1%, average 1.1%) (Figure 3b).

Comparison of α‐diversity indices among all heterospecific populations in the same site showed that there was no significant difference in Chao1 and Shannon indices between groups at S1, S2, S3 and S5, but the populations S4.Ap.a and S4.Cr.b at site 4 had a significant difference in the Chao1 indices (*p* = .0081) and Shannon indices (*p* = .0024) (Figure 3c, d; Table 2). In cases of conspecific populations at different sites, S4.Ap.a exhibited the highest level of α‐diversity than that of other allopatric *A. agrarius* populations, while S5.Ap.a showed the lowest level (Figure 3c,d; Table 2). The allopatric *C. barabensis* populations also showed different α‐diversity levels, with the S3.Cr.b holding higher Shannon indices than that of S4.Cr.b (*p* < .001) and higher Chao1 indices than that of S5.Cr.b (*p* < .001), and the S5.Cr.b had higher Shannon indices than that of S4.Cr.b (*p* < .001) (Figure 3c, d; Table 2). However, there were no significant differences in α‐diversity index between the two geographic populations of *R. norvegicus* (Figure 3c, d; Table 2). Together, these results suggested that species cohabiting within the same habitat tended to exhibit similar levels of α‐diversity due to the shared microbial pool, suggesting pronounced influence of the environment on the composition and diversity of animal gut microbiota.

**TABLE 2:** Intergroup Wilcoxon rank‐sum difference test for α‐diversity between conspecifics from the same habitat and heterospecifics from different habitats.

Group pair		Chao1		Shannon	
		Difference	*p* Value	Difference	*p* Value
Same site, heterospecific	S1.Ap.a‐S1.Ts.t	4.233	.5945	−2.344	.8320
	S2.Ap.a‐S2.Ra.n	−3.222	.6728	−13.056	.2204
	S3.Ap.a‐S3.Cr.b	−12.733	.1564	−19.533	.1185
	S4.Ap.a‐S4.Cr.b	22.175	.0081**	35.575	.0024**
	S5.Ap.a‐S5.Cr.b	−13.875	.1924	−24.250	.1024
	S5.Ap.a‐S5.Ra.n	−15.458	.1890	10.917	.5031
	S5.Cr.b‐S5.Ra.n	−1.583	.9046	35.167	.0583
Different sites, conspecific	S1.Ap.a‐S2.Ap.a	25.900	.0007***	25.267	.0159*
	S1.Ap.a‐S3.Ap.a	−9.767	.1690	2.833	.7730
	S1.Ap.a‐S4.Ap.a	−37.400	.0000***	5.400	.6159
	S1.Ap.a‐S5.Ap.a	32.025	.0002***	11.850	.3005
	S2.Ap.a‐S3.Ap.a	−35.667	.0000***	−22.433	.0179*
	S2.Ap.a‐S4.Ap.a	−63.300	.0000***	−19.867	.0564
	S2.Ap.a‐S5.Ap.a	6.125	.4386	−13.417	.2237
	S2.Ra.n‐S5.Ra.n	−6.111	.5963	10.556	.5109
	S3.Ap.a‐S4.Ap.a	−27.633	.0002***	2.567	.7938
	S3.Cr.b‐S4.Cr.b	7.275	.4613	57.675	.0001***
	S3.Ap.a‐S5.Ap.a	41.792	.0000***	9.017	.3929
	S3.Cr.b‐S5.Cr.b	40.650	.0007***	4.300	.7899
	S4.Ap.a‐S5.Ap.a	69.425	.0000***	6.450	.5722
	S4.Cr.b‐S5.Cr.b	33.375	.0022**	−53.375	.0005***

*Note*: Bold indicates significant differences in the compared groups (Ap.a: *Apodemus agrarius*, Cr.b: *Cricetulus barabensis*, Ts.t: *Tscherskia triton*, Ra.n: *Rattus norvegicus*).

*p < .05, **p < .01 and ***p < .001.

### 3.4 Comparison of gut microbiota β‐diversity between populations

**FIGURE 4:**
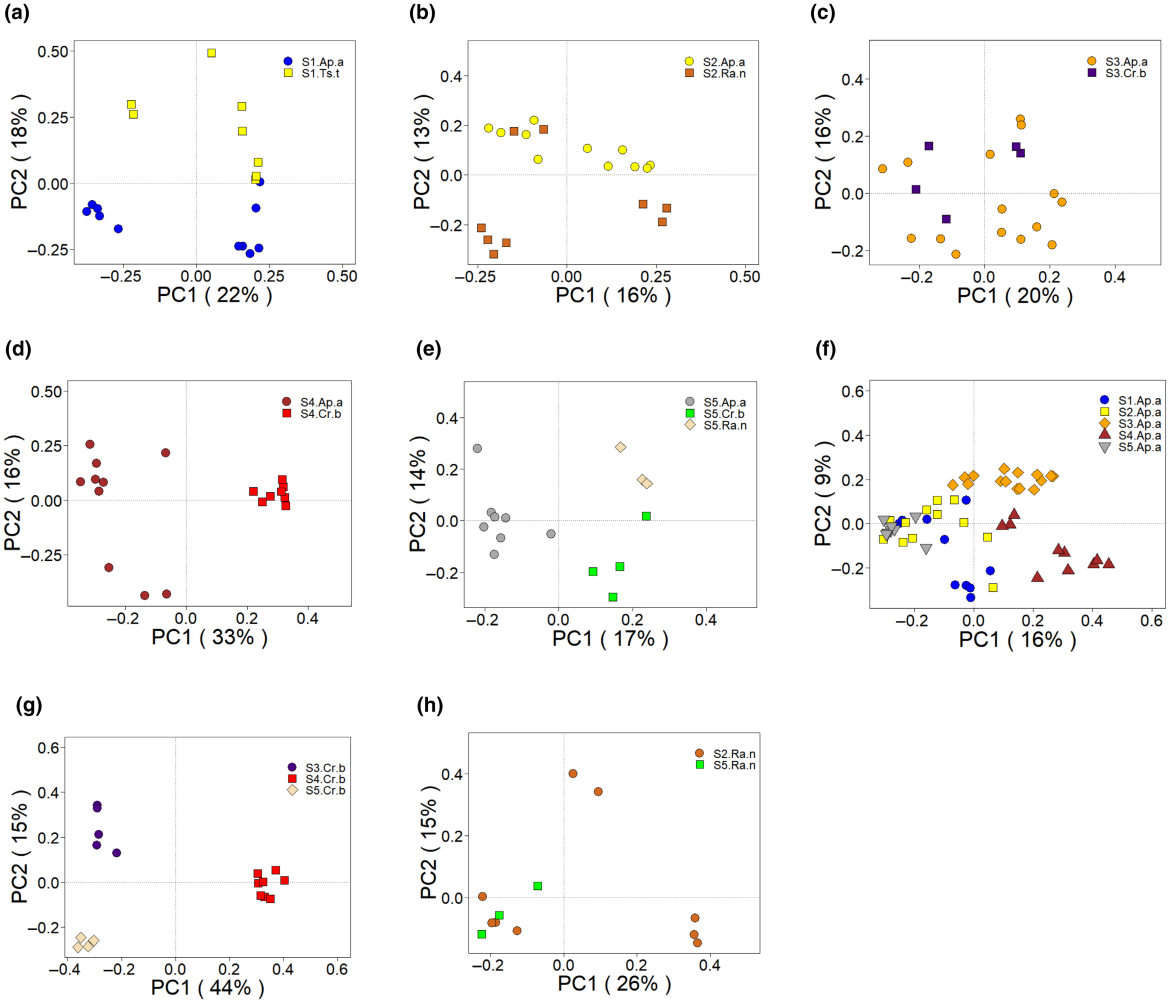
Clustering of gut microbial communities according to species identity and capture site. (a–e) Principle coordinate (PCoA) plots based on Bray–Curtis dissimilarity for heterospecific populations in different sites and conspecific populations in the same site (f–h) (Ap.a: *Apodemus agrarius*, Cr.b: *Cricetulus barabensis*, Ts.t: *Tscherskia triton*, Ra.n: *Rattus norvegicus*).

PCoA plots based on Bray–Curtis dissimilarity showed clear sample clustering not only by host species but also by capture site. The caecal samples from the same rodent species significantly clustered together (Figure 4a, b, d, e, Table 3), which indicated that the gut microbial compositions among different individuals of the same species were highly similar. However, the *A. agrarius* did not exhibit clear differentiation from the corresponding *C. barabensis* at S3 (Figure 4c) and S5 (Figure 4e), which indicated that in similar habitats, *A. agrarius* and *C. barabensis* shared a similar gut microbiota structure.

**TABLE 3:** Permutation multivariate analysis of variance (PERMANOVA) results for gut microbiota composition between different populations based on Bray–Curtis distance.

	Group pair	F.Model	*R* ^2^	Pr (>F)
Same site, heterospecific	S1.Ap.a‐S1.Ts.t	3.0755	.1532 (.8468)	.007**
	S2.Ap.a‐S2.Ra.n	2.6805	.12364 (.87636)	.02*
	S3.Ap.a‐S3.Cr.b	0.90782	.04801 (.95199)	.454
	S4.Ap.a‐S4.Cr.b	6.1005	.27603 (.72397)	.001**
	S5.Ap.a‐S5.Cr.b	1.5191	.13188 (.86812)	.136
	S5.Ap.a‐S5.Ra.n	3.6764	.29002 (.70998)	.005**
	S5.Ra.n‐S5.Cr.b	3.5229	.41334 (.58666)	.023*
Different sites, conspecific	S1.Ap.a‐S2.Ap.a	2.2012	.09915 (.90085)	.035*
	S1.Ap.a‐S3.Ap.a	5.8263	.20212 (.79788)	.001**
	S1.Ap.a‐S4.Ap.a	5.3174	.22804 (.77196)	.001**
	S1.Ap.a‐S5.Ap.a	2.972	.15665 (.84335)	.003**
	S2.Ap.a‐S3.Ap.a	4.2018	.14389 (.85611)	.001**
	S2.Ap.a‐S4.Ap.a	5.6334	.21977 (.78023)	.001**
	S2.Ap.a‐S5.Ap.a	3.3329	.15623 (.84377)	.005**
	S2.Ra.n‐S5.Ra.n	1.5217	.13207 (.86793)	.138
	S3.Ap.a‐S4.Ap.a	8.3219	.26569 (.73431)	.001**
	S3.Ap.a‐S5.Ap.a	14.014	.40024 (.59976)	.001**
	S3.Cr.b‐S4.Cr.b	11.87	.51903 (.48097)	.001**
	S3.Cr.b‐S5.Cr.b	7.8036	.52714 (.47286)	.005**
	S4.Ap.a‐S5.Ap.a	15.909	.49857 (.50143)	.001**
	S5.Cr.b‐S4.Cr.b	26.571	.72656 (.27344)	.003**

Abbreviations: Ap.a: *Apodemus agrarius*; Cr.b: *Cricetulus barabensis*; ns, not significant; Ra.n: *Rattus norvegicus*; Ts.t: *Tscherskia triton*.

*p < .05, **p < .01 and ***p < .001.

When comparing inter‐specific differences among their conspecific populations at the different sites, significant distinctions were observed within the five populations of *A. agrarius* (S1.Ap.a, S2.Ap.a, S3.Ap.a, S4.Ap.a and S5.Ap.a) (Figure 4f). Similarly, the three heterospecific *C. barabensis* populations (S3.Cr.b, S4.Cr.b and S5.Cr.b) showed a noticeable separation (Figure 4g). However, the two populations of *R. norvegicus* (S2.Ra.n and S5.Ra.n) appeared relatively dispersed, this might suggest that the within‐group differences for both groups were higher than the between‐group differences (Figure 4h).

**FIGURE 5:**
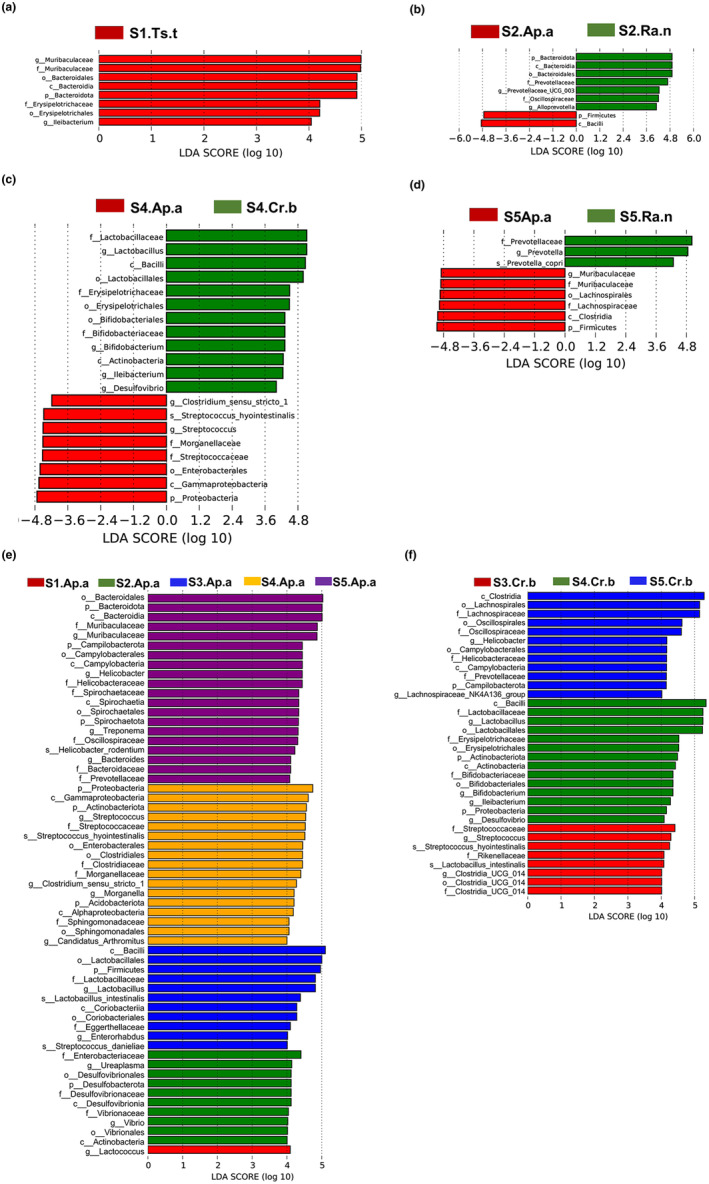
LEfSe analysis identified differential bacterial taxa between heterospecific populations in the same site (a–d) and between conspecific populations in the different sites (e, f). The LDA value distribution histogram shows species with LDA score above the set value (the default setting value is 4). That is, marker species are associated with significant differences between the groups. The length of the histogram represents the influence of the different species (LDA Score) (Ap.a: *Apodemus agrarius*, Cr.b: *Cricetulus barabensis*, Ts.t: *Tscherskia triton*, Ra.n: *Rattus norvegicus*).

Furthermore, the linear discriminant analysis effect size (LEfSe) noted that there were eight distinct taxa (LDA = 4) between the *A. agrarius and T. triton* population at S1, and all of these taxa were enriched within *T. triton* (Figure 5a). Nine different taxa (LDA = 4) between *A. agrarius* and *R. norvegicus* in S2, mainly Bacteroidetes and Firmicutes (Figure 5b), and in S4 (LDA = 4), the phylum of Proteobacteria and some potential pathogenic genera of bacteria, such as *Morganella* and *Streptococcus*, were enriched most within S4.Ap.a, while genera of *Lactobacillus* and *Bifidobacterium* that were considered probiotics were enriched in *C. barabensis* (Figure 5c). The main differences between *C. barabensis* and *R. norvegicus* in S5 (LDA = 4) were found in the Firmicutes phylum and the Muribaculaceae family (known as the S24‐7 family) (Figure 5d). Notably, no taxonomic differences were detected between *A. agrarius* and *C. barabensis* at S3. Interestingly, we observed an increase in the number of taxa when comparing the conspecific populations at different sites. Five isolated geographical populations of *A. agrarius* were discriminated by a total of 59 taxa (LDA = 4). These taxonomic distinctions were primarily delineated across 8 phyla (including Firmicutes, Actinobacteria and other core phyla), 9 classes (including Bacilli, Bacteroidia and others), 10 orders (including Lactobacillales, Bacteroidales, Enterobacterales, Spirochaetales, Clostridiales and more), 15 families (including Muribaculaceae, Streptococcaceae, Vibrionales, Lactobacillaceae, Enterobacteriaceae and others) and 12 genera (*Lactobacillus*, *Muribaculaceae*, *Streptococcus*, *Ureaplasma* and others) (Figure 5e). For allopatric speciation of *C. barabensis*, we detected 34 significantly distinct taxa (LDA = 4). These groups mainly encompassed 3 phyla (including Campilobacterota, Proteobacteria and Actinobacteriota), 4 classes (including Clostridia, Campylobacteria, Bacilli and Actinobacteria), 7 orders (including Lachnospirales, Oscillospirales, Campylobacterales, etc.), 10 families (including Lachnospiraceae, Oscillospiraceae, Prevotellaceae, Helicobacteraceae, etc.) and 8 genera (*Helicobacter*, *Lactobacillus*, *Bifidobacterium*, *Streptococcus*, etc.) (Figure 5f). However, no significant bacterial differences were detected at the taxonomic level between the two geographic populations S2.Ra.n and S5.Ra.n. The results showed heterospecific rodents living in the same site converged somewhat in the gut microbiota structure than that of conspecific rodents living in different sites.

### 3.5 Association of environmental and host factors with microbial diversity and composition

**FIGURE 6:**
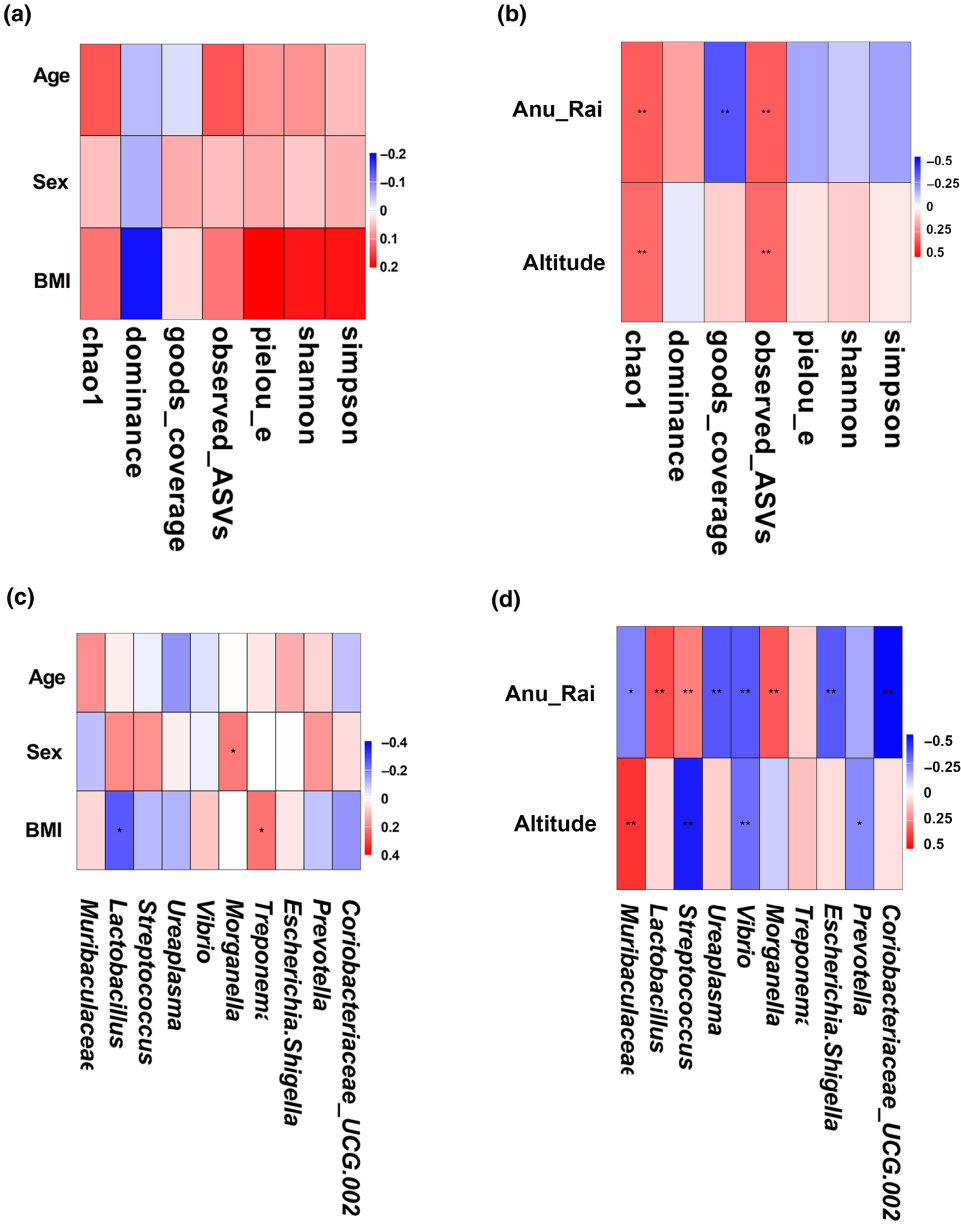
Spearman analysis of the correlation between host‐related internal factors and external factors with gut microbiota diversity changes, as well as correlation analysis with genus‐level abundance changes. (a) Host‐related internal factors (age, sex and BMI). (b) External factors (Anu_Rai: annual average precipitation, Altitude: altitude). (c) Correlation between host‐related internal factors and the genus‐level structural variation of gut microbiota. (d) Correlation between external factors and the genus‐level structural variation of gut microbiota.

Spearman analysis showed that host factors such as age, sex and BMI had minimal effects on the variation in gut microbiota abundance (Figure 6a). In contrast, external environmental factors displayed distinct associations; altitude exhibited significant positive correlations with the Chao1 index (*R* ^2^ = .293, *p* = .002) and the observed ASV index (*R* ^2^ = .294, *p* = .002). Meanwhile, the annual average precipitation (Anu_Rai) had an up‐regulating effect on the Chao1 index (*R* ^2^ = .320, *p* < .001), dominance index (*R* ^2^ = .183, *p* = .06), observed ASV index (*R* ^2^ = .319, *p* < .001) and had a down‐regulating effect on the goods_coverage index (*R* ^2^ = −0.334, *p* < .001), pielou_e index (*R* ^2^ = −.173, *p* = .07) and Simpson index (*R* ^2^ = −.184, *p* = .06) (Figure 6b). These results demonstrated that host factors had minimal impact, whereas external environmental factors exhibited significant associations with the diversity of the gut microbiota in the investigated cohort of small mammals.

**FIGURE 7:**
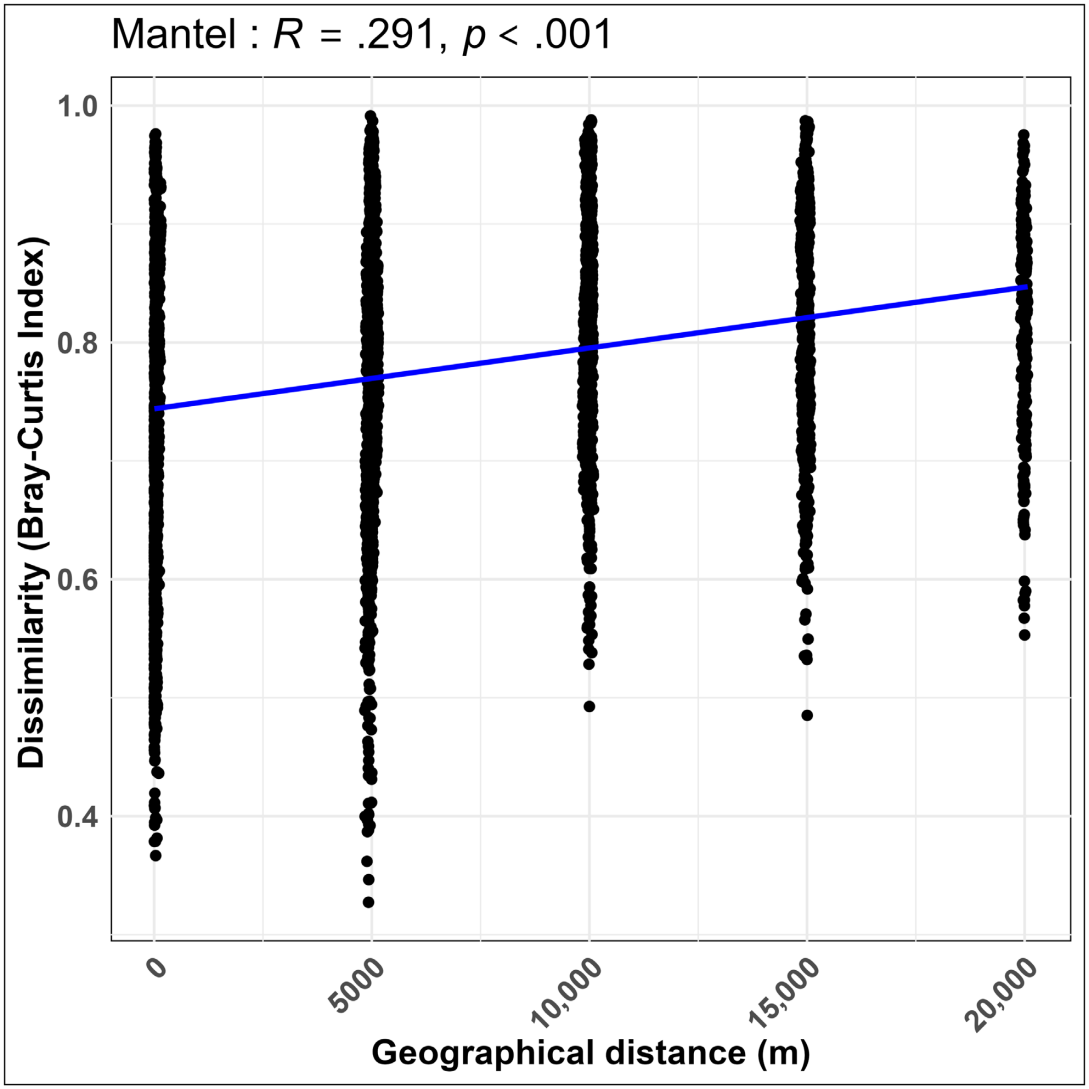
Spearman analysis of the dissimilarity among microbiota and geographic distance. Linear regression of sample geographical distance against the Bray–Curtis dissimilarity distance matrix, depicted by a straight line in academic terms.

Furthermore, correlation analysis between core genera and these influencing factors revealed significant associations. Regarding the host‐related factors, BMI was significantly negatively correlated with *Lactobacillus* (*R* ^2^ = −.26, *p* = .002) and significantly positively correlated with *Treponema* (*R* ^2^ = .22, *p* = .002), whereas sex exhibited a significant positive correlation with *Morganella* (*R* ^2^ = 0.21, *p* = .002) (Figure 6c). In the case of external host factors, annual average precipitation (Anu_Rai) demonstrated a significant positive correlation with *Lactobacillus* (*R* ^2^ = 0.408, *p* < .001), *Streptococcus* (*R* ^2^ = .321, *p* = .009) and *Morganella* (*R* ^2^ = .39, *p* < .001), while displaying a significant negative correlation with *Muribaculaceae* (*R* ^2^ = −.318, *p* = .01), *Ureaplasma* (*R* ^2^ = −.369, *p* < .001), *Vibrio* (*R* ^2^ = −.359, *p* < .001), *Treponema* (*R* ^2^ = −.405, *p* < .001) and *Prevotella* (*R* ^2^ = −.577, *p* < .001). Altitude was significantly positively correlated with *Muribaculaceae* (*R* ^2^ = .371, *p* < .001) and significantly negatively correlated with *Streptococcus* (*R* ^2^ = −.415, *p* < .001) and *Vibrio* (*R* ^2^ = −.297, *p* = .003) (Figure 6d). Additionally, we observed a significant positive correlation between the gut microbiota dissimilarity and spatial distance (*R* ^2^ = .291, *p* < .001) (Figure 7). In short, the correlation analyses underscored intricate associations between core genera and both host‐related and external environmental factors and emphasized the multifaceted interplay shaping the gut microbiota composition in the studied small mammals. The positive correlation between microbiota dissimilarity and spatial distance further suggested potential spatial influences on microbial community structure.

## 4 DISCUSSION

This study provides a comprehensive analysis of the gut microbiota of rodent populations, shedding light on diversity, phylogenetic structure and the influential factors that affect microbial composition. We found the α‐diversity among different populations converged if they shared a microbial pool (inhabited the same site), while they still possessed certain β‐diversity differences. This suggests that the environment determines the specific bacterial species present in the gut, whereas the host regulates the abundance of these species. In addition, significant microbial differentiation was observed among the different geographic populations of *A. agrarius* and *C. barabensis*. Furthermore, as the geographic distance increased, the microbial dissimilarity among these distinct populations increased. However, the gut microbiota of *R. norvegicus* appeared to be minimally influenced by geographic distance, likely because of their strong dispersal ability and omnivorous nature.

Recent studies have provided evidence of the formation of species between mammals and their gut microbiota (Amato et al., 2016). Compared to clinical studies, microbial research targeting wild animals was limited. Several studies have focused on single populations of a single host species. In our study, we conducted a large‐scale geographical survey of natural rodent populations from the farming–pastoral ecotone in northern China (Chen et al., 2008). Compared to clinical studies, microbial research targeting wild animals was limited. Several studies have focused on single populations of a single host species. In our study, we conducted a large‐scale geographical survey of natural rodent populations from the farming–pastoral ecotone in northern China (Han et al., 2018). Therefore, we hypothesized that the gut microbiota of rodents inhabiting the farming–pastoral ecotone was highly responsive to environmental changes. To substantiate this hypothesis, we designed sampling sites with distances exceeding 30 km to mitigate the impact of inter‐species gene flow.

Therefore, we hypothesized that the gut microbiota of rodents inhabiting the farming–pastoral ecotone was highly responsive to environmental changes. To substantiate this hypothesis, we designed sampling sites with distances exceeding 30 km to mitigate the impact of inter‐species gene flow (Chen et al., 2020; Montoya‐Ciriaco et al., 2020; Suzuki et al., 2019). Similar patterns in geographical locations further underline the role of the environment in microbial composition. A plausible explanation is that the gut microbiota composition of mammals is influenced by the geographical distance between locations, owing to bacterial dispersal constraints (Moeller et al., 2017). Within smaller geographical ranges, a more likely explanation is the profound impact of shared environmental resources (Lavrinienko et al., 2021).

Following the QIIME pipeline, we found that the gut microbiota of wild rodents was comparable to that of other mammals, with two major phyla: Firmicutes and Bacteroidetes (Amato et al., 2016; Benson et al., 2010; Ross et al., 2018). Our results showed that the relative abundance of Firmicutes was higher than that of Bacteroidetes in *A. agrarius*, *C. barabensis* and *R. norvegicus*, whereas that of Bacteroidetes was higher than that of Firmicutes in *T. triton*. This underscores the potential influence of dietary factors on microbial composition (Zoelzer et al., 2021). *A. agrarius*, *C. barabensis* and *R. norvegicus* are not strict herbivores, such as squirrels and lemurs (Greene et al., 2020; Liu et al., 2020; Riofrio‐Lazo & Paez‐Rosas, 2015; Shi et al., 2017), whereas *T. triton* is herbivorous, occasionally incorporating insects into its diet (Xiong et al., 2009). Firmicutes are a common type of bacteria known for their ability to degrade various complex organic compounds and may be more abundant in omnivorous animals to facilitate the digestion and absorption of a diverse range of foods (Guindo et al., 2020). Bacteroidetes, which are typically abundant in the gut of herbivorous animals, are known for their ability to break down plant cellulose and polysaccharides present in plant materials, aiding animals in the digestion of fibre to acquire energy and nutrients (Clauss et al., 2020; Fu et al., 2021). Why some bacterial groups are more host specific than others remains an interesting open question. One possibility is that some bacteria are more amenable to host selection via immunity (Benson et al., 2010; Kurilshikov et al., 2017). Such biological differences warrant further investigation as potential host specificity mediators.

By comparing microbial community variations between heterospecific and conspecific populations, we revealed that heterospecific populations within the same habitat had a higher conserved bacteria abundance, whereas conspecific populations in different habitats had a lower conserved bacteria abundance. Some other research also indicated that shared habitats significantly promote α‐diversity convergence among conspecific symbiotic populations within the same habitat (Grieneisen et al., 2019; Grond et al., 2020). Additionally, our results suggested that geographical factors play a species‐specific role in shaping the α‐diversity of host gut microbiota, with *A. agrarius* and *C. barabensis* populations being more susceptible to the influence of geographical factors, whereas this influence was less pronounced in *R. norvegicus* populations. We found that there was no significant difference in β‐diversity of gut microbiota between the *A. agrarius* and *C. barabensis* at site 3, indicating a higher similarity in gut microbiota between these two species in a homogenized environment, which could be disadvantageous as it may intensify inter‐specific competition between the two species (Anders et al., 2022; Shaner & Ke, 2022). In addition, the observed within‐group differences being higher than between‐group differences in certain cases (such as *R. norvegicus*) suggests unique microbial adaptations within specific populations (He et al., 2020).

Correlation analyses between environmental and host factors and microbial diversity revealed interesting associations. Although we did not observe a statistically significant influence of sex on the detected variations, this aligned with findings in research involving small wild mammals, such as woodrat, vole and house mice (Goertz et al., 2019; Lin et al., 2020; Weinstein et al., 2021). This consistent pattern suggests that fluctuations in the gut microbiota resulting from natural environmental dynamics may overshadow differences attributable to host factors such as sex and age. However, in larger mammals, particularly those with a significant relative body size, such as vertebrates, the impact of sex tends to be more conspicuous (Adriansjach et al., 2020; Bjork et al., 2022). Additionally, controlled experiments under laboratory conditions, where environmental variables were meticulously regulated, have emphasized and demonstrated the presence of sex‐based disparities (Kohl, Dearing, & Bordenstein, 2018a; Screven & Dent, 2018). Furthermore, we discovered that higher altitudes were positively correlated with microbial diversity indices, indicating potential adaptation to high‐altitude environments (Li et al., 2020; Quagliariello et al., 2019; Suzuki et al., 2019). Moreover, the impact of annual average precipitation on microbial diversity suggests that environmental factors significantly influence gut microbiota. The taxonomic differences identified between populations and their correlations with environmental factors provide valuable insights into potential allopatric speciation events (Baral et al., 2018; Khakisahneh et al., 2020). Distinct taxa enriched within specific populations indicate the potential adaptation and co‐evolution of the gut microbiota with their host species in different geographic locations (Suzuki et al., 2020).

Our findings have broad implications for understanding the dynamics of gut microbiota in rodent populations. The highlighted influence of the observed environmental factors underscores the necessity of considering habitat and diet in future investigations. Moreover, a deeper understanding of the ecological and evolutionary factors shaping the gut microbiota could be achieved by incorporating more host‐associated information, such as inter‐individual social contacts, kinship, social hierarchy, lineage differentiation and other related aspects.

In conclusion, this study provides a comprehensive understanding of the gut microbiota in rodent populations and elucidates the intricate interplay among host identity, environment and microbial communities. We revealed that the dominant role of species identity in the environment may not be universal, at least to a certain extent. As the investigation scope broadened, the explanatory power of spatial distance concerning the variations in the host's gut microbiota surpassed that of host identity. These findings contribute to a broader understanding of gut microbiota dynamics and their potential implications for ecology, evolution and health.

## AUTHOR CONTRIBUTIONS

**Yongzhen Wu:** Conceptualization (lead); data curation (equal); formal analysis (lead); investigation (lead); methodology (equal); project administration (equal); writing – original draft (equal). **Taoxiu Zhou:** Data curation (supporting); formal analysis (equal); investigation (equal); visualization (supporting); writing – original draft (supporting). **Chen Gu:** Conceptualization (equal); data curation (supporting); formal analysis (supporting); funding acquisition (supporting); investigation (supporting); methodology (supporting); project administration (supporting); writing – original draft (supporting). **Baofa Yin:** Conceptualization (supporting); data curation (supporting); formal analysis (supporting); project administration (supporting); supervision (supporting). **Shengmei Yang:** Conceptualization (supporting); formal analysis (supporting); methodology (supporting); project administration (supporting); supervision (supporting). **Yunzneg Zhang:** Data curation (supporting); formal analysis (supporting); methodology (supporting); supervision (supporting); writing – original draft (supporting). **Ruiyong Wu:** Conceptualization (supporting); formal analysis (supporting); investigation (supporting); methodology (supporting); writing – original draft (supporting). **Wanhong Wei:** Conceptualization (lead); funding acquisition (lead); investigation (lead); methodology (lead); supervision (lead).

## FUNDING INFORMATION

National Natural Science Foundation of China, Grant/Award Number: 31770422 and 31971429.

## CONFLICT OF INTEREST STATEMENT

The authors declare that they have no competing interests for the publication of this study.

## Supporting information


Data S1.

## Data Availability

Molecular sequence data have been deposited in the NCBI Sequence Read Archive (SRA) database (accession number PRJNA1019510).
