# Supplementary material for: Geographical distribution and species variation of gut microbiota in small rodents from the agro‐pastoral transition ecotone in northern China
Source: Ecol Evol. 2024 Mar 11;14(3):e11084. doi: 10.1002/ece3.11084 (PMC10926059; doi:10.1002/ece3.11084)
Supplement: Supplementary file 1 — Data S1. [file ECE3-14-e11084-s001.doc]

**Supplementary Information for**

**Geographical Distribution and Species Variation of Gut Microbiota in Small Rodents from the Agro-pastoral Transition Ecotone in Northern China**

**Table S1 | Sample Information.** The age classification of the ***A. agrarius*** was based on the following criteria: Juvenile group (I), body weight ≤ 12.99g; Subadult group (Ⅱ), body weight 13.00-16.99g; Adult group I (Ⅲ), body weight 17.00-20.99g; Adult group II (Ⅳ), body weight 21.00-25.99g; Senile group (V), body weight ≥ 26.0g. The age classification of the ***T. triton*** was based on the following criteria: Juvenile group (Ⅰ), body weight ≤ 35g; Subadult group (Ⅱ), body weight 35.1-79g; Adult group Ⅰ (Ⅲ), body weight 79.1-123g; Adult group Ⅱ (Ⅳ), body weight 123-187g; Senile group (Ⅴ), body weight ≥ 187g. The age classification of the ***C. barabensis*** was based on the following criteria: Juvenile group (Ⅰ), body weight ≤ 11.0g; Subadult group (Ⅱ), body weight 11.1-15g; Adult group Ⅰ (Ⅲ), body weight ≥ 15.1-19g; Adult group Ⅱ(Ⅳ), body weight 19.1- 23g; Senile group (Ⅴ), body weight ≥ 23.1g. The age classification of the ***R. norvegicus*** was based on the following criteria: Juvenile group (I), body weight ≤ 60g; Subadult group (Ⅱ), body weight 60.1-110.0g; Adult group I (Ⅲ), body weight 110.1-160.0g; Adult group II (Ⅳ), body weight 160.1-210.0g; Senile group (V), body weight ≥ 210.1g.

| **sample ID** | **species** | **localities** | **Location number** | **sex** | **weight** | **BMI** | **carcass weight** | **age** | **date** |
| --- | --- | --- | --- | --- | --- | --- | --- | --- | --- |
| A.AP.6 | *A. agrarius* | DS | S1 | female | 24.97 | 0.00338 | 17.171 | Ⅲ | Jul-20 |
| A.AP.9 | *A. agrarius* | DS | S1 | female | 29.38 | 0.00319 | 17.303 | Ⅲ | Jul-20 |
| A.AP.7 | *A. agrarius* | DS | S1 | female | 30.54 | 0.00331 | 19.431 | Ⅲ | Jul-20 |
| A.AP.11 | *A. agrarius* | DS | S1 | male | 31.2 | 0.00442 | 21.064 | Ⅳ | Jul-20 |
| A.AP.3 | *A. agrarius* | DS | S1 | male | 31.16 | 0.00283 | 22.010 | Ⅳ | Jul-20 |
| A.AP.8 | *A. agrarius* | DS | S1 | female | 34.45 | 0.00265 | 22.605 | Ⅳ | Jul-20 |
| A.AP.4 | *A. agrarius* | DS | S1 | female | 33.94 | 0.00361 | 22.763 | Ⅳ | Jul-20 |
| A.AP.2 | *A. agrarius* | DS | S1 | female | 41.48 | 0.00384 | 24.044 | Ⅳ | Jul-20 |
| A.AP.1 | *A. agrarius* | DS | S1 | male | 40.51 | 0.00367 | 24.432 | Ⅳ | Jul-20 |
| A.AP.5 | *A. agrarius* | DS | S1 | male | 35.72 | 0.00388 | 27.541 | V | Jul-20 |
| A.TS.5 | *T. triton* | DS | S1 | female | 47.86 | 0.00410 | 35.384 | Ⅱ | Jul-20 |
| A.AP.10 | *T. triton* | DS | S1 | male | 66.87 | 0.00384 | 45.904 | Ⅱ | Jul-20 |
| A.TS.1 | *T. triton* | DS | S1 | male | 79.65 | 0.00315 | 48.770 | Ⅲ | Jul-20 |
| A.TS.3 | *T. triton* | DS | S1 | male | 77.99 | 0.00404 | 53.405 | Ⅲ | Jul-20 |
| A.TS.7 | *T. triton* | DS | S1 | female | 75.39 | 0.00390 | 53.954 | Ⅲ | Jul-20 |
| A.TS.6 | *T. triton* | DS | S1 | male | 97.48 | 0.00411 | 63.668 | Ⅳ | Jul-20 |
| A.TS.4 | *T. triton* | DS | S1 | female | 124.82 | 0.00570 | 73.840 | Ⅳ | Jul-20 |
| A.TS.8 | *T. triton* | DS | S1 | male | 126.13 | 0.00421 | 94.171 | Ⅳ | Jul-20 |
| A.TS.2 | *T. triton* | DS | S1 | female | 125.67 | 0.00430 | 92.115 | Ⅳ | Jul-20 |
| B.TS.2 | *T. triton* | WB | S2 | male | 17.56 | 0.00237 | 11.385 | I | Jul-20 |
| B.TS.1 | *T. triton* | WB | S2 | female | 30.8 | 0.00334 | 21.443 | I | Jul-20 |
| B.AP.5 | *A. agrarius* | WB | S2 | male | 22.81 | 0.00295 | 15.003 | Ⅱ | Jul-20 |
| B.AP.7 | *A. agrarius* | WB | S2 | male | 23.63 | 0.00256 | 16.039 | Ⅱ | Jul-20 |
| B.AP.1 | *A. agrarius* | WB | S2 | male | 26.22 | 0.00303 | 18.158 | Ⅲ | Jul-20 |
| B.AP.8 | *A. agrarius* | WB | S2 | female | 31.13 | 0.00305 | 18.581 | Ⅲ | Jul-20 |
| B.AP.6 | *A. agrarius* | WB | S2 | male | 30.58 | 0.00361 | 20.655 | Ⅲ | Jul-20 |
| B.AP.2 | *A. agrarius* | WB | S2 | female | 34.03 | 0.00393 | 20.888 | Ⅲ | Jul-20 |
| B.AP.3 | *A. agrarius* | WB | S2 | male | 34.19 | 0.00395 | 22.945 | Ⅳ | Jul-20 |
| B.AP.4 | *A. agrarius* | WB | S2 | male | 34.58 | 0.00308 | 24.644 | Ⅳ | Jul-20 |
| B.AP.12 | *A. agrarius* | WB | S2 | female | 35.46 | 0.00369 | 19.847 | Ⅲ | Jul-20 |
| B.AP.11 | *A. agrarius* | WB | S2 | male | 35.43 | 0.00347 | 22.671 | Ⅳ | Jul-20 |
| B.AP.9 | *A. agrarius* | WB | S2 | male | 36.54 | 0.00358 | 23.876 | Ⅳ | Jul-20 |
| B.AP.10 | *A. agrarius* | WB | S2 | male | 36.42 | 0.00343 | 26.481 | V | Jul-20 |
| B.RA.2 | *R. norvegicus* | WB | S2 | male | 31.43 | 0.00308 | 20.626 | I | Jul-20 |
| B.RA.7 | *R. norvegicus* | WB | S2 | male | 59.26 | 0.00325 | 41.815 | I | Jul-20 |
| B.RA.6 | *R. norvegicus* | WB | S2 | female | 105.66 | 0.00457 | 69.910 | Ⅱ | Jul-20 |
| B.RA.4 | *R. norvegicus* | WB | S2 | male | 115.49 | 0.00493 | 85.704 | Ⅲ | Jul-20 |
| B.RA.3 | *R. norvegicus* | WB | S2 | male | 120.63 | 0.00427 | 87.941 | Ⅳ | Jul-20 |
| B.RA.8 | *R. norvegicus* | WB | S2 | male | 146.47 | 0.00478 | 104.300 | Ⅳ | Jul-20 |
| B.RA.5 | *R. norvegicus* | WB | S2 | male | 146.5 | 0.00468 | 107.812 | Ⅳ | Jul-20 |
| B.RA.9 | *R. norvegicus* | WB | S2 | female | 164.93 | 0.00457 | 116.313 | Ⅳ | Jul-20 |
| B.RA.1 | *R. norvegicus* | WB | S2 | male | 36.67 | 0.00352 | 25.252 | I | Jul-20 |
| B.AP.13 | *A. agrarius* | WB | S2 | female | 28.71 | 0.00199 | 17.437 | Ⅲ | Jul-20 |
| B.AP.16 | *A. agrarius* | WB | S2 | female | 32.16 | 0.00261 | 19.000 | Ⅲ | Jul-20 |
| B.AP.15 | *A. agrarius* | WB | S2 | female | 26.51 | 0.00223 | 20.922 | Ⅲ | Jul-20 |
| B.AP.14 | *A. agrarius* | WB | S2 | female | 30.95 | 0.00343 | 21.602 | Ⅳ | Jul-20 |
| B.AP.23 | *A. agrarius* | WB | S2 | male | 26.53 | 0.00241 | 18.518 | Ⅲ | Jul-20 |
| B.AP.18 | *A. agrarius* | WB | S2 | female | 27.68 | 0.00266 | 18.699 | Ⅲ | Jul-20 |
| B.AP.17 | *A. agrarius* | WB | S2 | female | 30 | 0.00272 | 19.815 | Ⅲ | Jul-20 |
| B.AP.21 | *A. agrarius* | WB | S2 | female | 27.73 | 0.00242 | 20.081 | Ⅲ | Jul-20 |
| B.AP.22 | *A. agrarius* | WB | S2 | female | 34.7 | 0.00253 | 23.442 | Ⅳ | Jul-20 |
| B.AP.24 | *A. agrarius* | WB | S2 | male | 40.77 | 0.00298 | 26.034 | V | Jul-20 |
| B.AP.20 | *A. agrarius* | WB | S2 | male | 38.91 | 0.00241 | 26.943 | V | Jul-20 |
| B.AP.19 | *A. agrarius* | WB | S2 | female | 19.42 | 0.00215 | 13.024 | Ⅱ | Jul-20 |
| C.CR.1 | *C. barabensis* | MZ | S3 | female | 19.95 | 0.00304 | 12.505 | I | Aug-20 |
| C.CR.3 | *C. barabensis* | MZ | S3 | female | 31.3 | 0.00362 | 20.084 | V | Aug-20 |
| C.CR.5 | *C. barabensis* | MZ | S3 | female | 35.39 | 0.00392 | 20.499 | V | Aug-20 |
| C.CR.2 | *C. barabensis* | MZ | S3 | female | 44.78 | 0.00422 | 26.583 | V | Aug-20 |
| C.CR.4 | *R. norvegicus* | MZ | S3 | male | 37.38 | 0.00366 | 28.410 | V | Aug-20 |
| C.RA.1 | *R. norvegicus* | MZ | S3 | male | 171.71 | 0.00554 | 127.652 | Ⅳ | Aug-20 |
| C.TS.1 | *T. triton* | MZ | S3 | male | 59.28 | 0.00379 | 40.838 | Ⅱ | Aug-20 |
| C.AP.1 | *A. agrarius* | MZ | S3 | female | 27.97 | 0.00338 | 16.400 | Ⅱ | Aug-20 |
| C.AP.3 | *A. agrarius* | MZ | S3 | female | 29.51 | 0.00334 | 18.868 | Ⅲ | Aug-20 |
| C.AP.2 | *A. agrarius* | MZ | S3 | male | 28.05 | 0.00346 | 19.029 | Ⅲ | Aug-20 |
| C.AP.15 | *A. agrarius* | MZ | S3 | male | 33.59 | 0.00364 | 21.181 | Ⅳ | Aug-20 |
| C.AP.5 | *A. agrarius* | MZ | S3 | female | 35.99 | 0.00339 | 22.097 | Ⅳ | Aug-20 |
| C.AP.4 | *A. agrarius* | MZ | S3 | female | 38.22 | 0.00294 | 23.921 | Ⅳ | Aug-20 |
| C.AP.14 | *A. agrarius* | MZ | S3 | male | 44.22 | 0.00340 | 31.226 | V | Aug-20 |
| C.AP.9 | *A. agrarius* | MZ | S3 | male | 28.42 | 0.00268 | 19.454 | Ⅲ | Aug-20 |
| C.AP.12 | *A. agrarius* | MZ | S3 | male | 30.18 | 0.00308 | 19.692 | Ⅲ | Aug-20 |
| C.AP.11 | *A. agrarius* | MZ | S3 | female | 29.91 | 0.00311 | 20.552 | Ⅲ | Aug-20 |
| C.AP.8 | *A. agrarius* | MZ | S3 | female | 34.8 | 0.00334 | 20.955 | Ⅲ | Aug-20 |
| C.AP.13 | *A. agrarius* | MZ | S3 | female | 30.7 | 0.00320 | 21.917 | Ⅳ | Aug-20 |
| C.AP.10 | *A. agrarius* | MZ | S3 | male | 37.84 | 0.00331 | 24.339 | Ⅳ | Aug-20 |
| C.AP.6 | *A. agrarius* | MZ | S3 | male | 39.7 | 0.00300 | 26.189 | V | Aug-20 |
| C.AP.7 | *A. agrarius* | MZ | S3 | male | 37.37 | 0.00326 | 26.941 | V | Aug-20 |
| D.AP.10 | *A. agrarius* | JM | S4 | male | 21.84 | 0.00341 | 15.431 | Ⅱ | Aug-20 |
| D.AP.4 | *A. agrarius* | JM | S4 | male | 31.22 | 0.00283 | 20.163 | Ⅲ | Aug-20 |
| D.AP.7 | *A. agrarius* | JM | S4 | female | 35.89 | 0.00389 | 21.459 | Ⅳ | Aug-20 |
| D.AP.3 | *A. agrarius* | JM | S4 | male | 32.89 | 0.00350 | 21.713 | Ⅳ | Aug-20 |
| D.AP.6 | *A. agrarius* | JM | S4 | male | 33.55 | 0.00282 | 22.506 | Ⅳ | Aug-20 |
| D.AP.9 | *A. agrarius* | JM | S4 | female | 35.19 | 0.00345 | 22.762 | Ⅳ | Aug-20 |
| D.AP.5 | *A. agrarius* | JM | S4 | male | 31.41 | 0.00296 | 24.928 | Ⅳ | Aug-20 |
| D.AP.8 | *A. agrarius* | JM | S4 | female | 36.74 | 0.00327 | 24.963 | Ⅳ | Aug-20 |
| D.CR.8 | *C. barabensis* | JM | S4 | female | 17.99 | 0.00274 | 13.336 | Ⅲ | Aug-20 |
| D.CR.7 | *C. barabensis* | JM | S4 | female | 21.1 | 0.00443 | 14.271 | Ⅳ | Aug-20 |
| D.CR.6 | *C. barabensis* | JM | S4 | female | 22.37 | 0.00387 | 14.606 | Ⅳ | Aug-20 |
| D.CR.2 | *C. barabensis* | JM | S4 | male | 23.37 | 0.00259 | 17.150 | V | Aug-20 |
| D.CR.5 | *C. barabensis* | JM | S4 | female | 25.9 | 0.00334 | 18.090 | V | Aug-20 |
| D.CR.3 | *C. barabensis* | JM | S4 | male | 32.67 | 0.00256 | 21.650 | V | Aug-20 |
| D.CR.4 | *C. barabensis* | JM | S4 | male | 32.78 | 0.00303 | 22.029 | V | Aug-20 |
| D.CR.1 | *C. barabensis* | JM | S4 | male | 40.99 | 0.00333 | 26.965 | V | Aug-20 |
| D.RA.1 | *R. norvegicus* | JM | S4 | female | 228.18 | 0.00499 | 175.440 | Ⅲ | Aug-20 |
| D.AP.1 | *A. agrarius* | JM | S4 | female | 25.58 | 0.00354 | 18.321 | Ⅲ | Aug-20 |
| D.AP.2 | *A. agrarius* | JM | S4 | male | 34.54 | 0.00285 | 24.065 | Ⅳ | Aug-20 |
| E.CR.2 | *C. barabensis* | DT | S5 | female | 26.61 | 0.00314 | 18.046 | V | Aug-20 |
| E.CR.4 | *C. barabensis* | DT | S5 | male | 32.05 | 0.00355 | 19.566 | V | Aug-20 |
| E.CR.3 | *C. barabensis* | DT | S5 | male | 30.8 | 0.00364 | 20.337 | V | Aug-20 |
| E.CR.1 | *C. barabensis* | DT | S5 | male | 32.84 | 0.00424 | 21.792 | V | Aug-20 |
| E.AP.3 | *A. agrarius* | DT | S5 | male | 23.65 | 0.00268 | 14.506 | Ⅱ | Aug-20 |
| E.AP.4 | *A. agrarius* | DT | S5 | male | 27.08 | 0.00306 | 16.675 | Ⅱ | Aug-20 |
| E.AP.2 | *A. agrarius* | DT | S5 | male | 31.1 | 0.00331 | 20.044 | Ⅲ | Aug-20 |
| E.AP.1 | *A. agrarius* | DT | S5 | male | 32.24 | 0.00310 | 20.781 | Ⅲ | Aug-20 |
| E.AP.8 | *A. agrarius* | DT | S5 | male | 29.99 | 0.00277 | 21.389 | Ⅳ | Aug-20 |
| E.AP.7 | *A. agrarius* | DT | S5 | male | 36.39 | 0.00312 | 22.226 | Ⅳ | Aug-20 |
| E.AP.5 | *A. agrarius* | DT | S5 | male | 34.4 | 0.00331 | 24.122 | Ⅳ | Aug-20 |
| E.AP.6 | *A. agrarius* | DT | S5 | female | 47.93 | 0.00461 | 28.843 | V | Aug-20 |
| E.RA.2 | *R. norvegicus* | DT | S5 | male | 107.21 | 0.00452 | 78.532 | Ⅱ | Aug-20 |
| E.RA.3 | *R. norvegicus* | DT | S5 | female | 141.78 | 0.00515 | 104.737 | Ⅳ | Aug-20 |
| E.RA.1 | *R. norvegicus* | DT | S5 | male | 161.86 | 0.00483 | 120.102 | Ⅳ | Aug-20 |

**Table S2 | Geographical information and partial meteorological data for five survey locations. Data were downloaded from the China National Meteorological Information Center (https: //data.cma.cn)**

| **Location number** | **localities** | **longitude** | **latitude** | **altitude** | **annual average temperature**  **(℃)** | **annual rainfall（mm）** |
| --- | --- | --- | --- | --- | --- | --- |
| S1 | DS | 45.5761 | 126.4849 | 182 | 4.67 | 656.47 |
| S2 | WB | 45.8685 | 126.3661 | 120 | 4.87 | 612.28 |
| S3 | MZ | 45.4973 | 125.3094 | 124 | 5.44 | 541.28 |
| S4 | JM | 45.5935 | 124.3706 | 139 | 5.68 | 490.64 |
| S5 | DT | 46.0079 | 124.8794 | 132 | 5.17 | 534.52 |

**Table S3 |** Species and Quantity of rodent captured at each site for which the gut microbiota was characterized. The groups that sample size was less than 3 would not be analyzed.

| **Species** | **Abbreviation** | **Site** | | | | |
| --- | --- | --- | --- | --- | --- | --- |
|  |  | **S1** | **S2** | **S3** | **S4** | **S5** |
| *Apodemus agrarius* | Ap.a | 10 | 24 | 15 | 10 | 8 |
| *Cricetulus barabensis* | Cr.b | 0 | 0 | 5 | 8 | 4 |
| *Tscherskia triton* | Ts.t | 9 | 2 | 1 | 0 | 0 |
| *Rattus norvegicus* | Ra.n | 0 | 9 | 1 | 1 | 3 |

**Table S4 | Annotation of all ASVs was performed based on the Silva bacterial database.** The table presents the quantity at different taxonomic levels.

| **Levels** | **Number** |
| --- | --- |
| Phylum | 36 |
| Class | 85 |
| Order | 183 |
| Family | 303 |
| Genus | 529 |

**
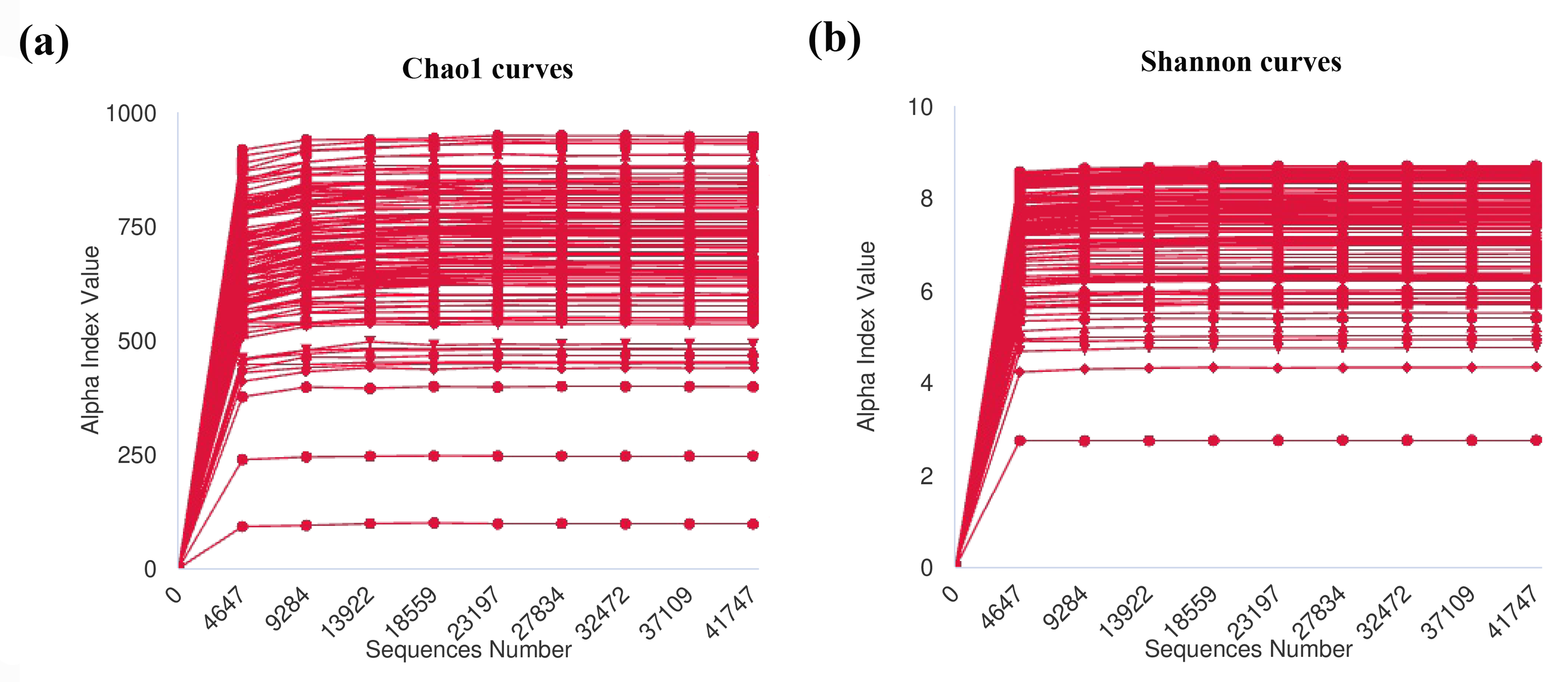
**

**Figure S1 | Rarefaction curves, coverage, and results of Chao1 and Shannon tests.**

**
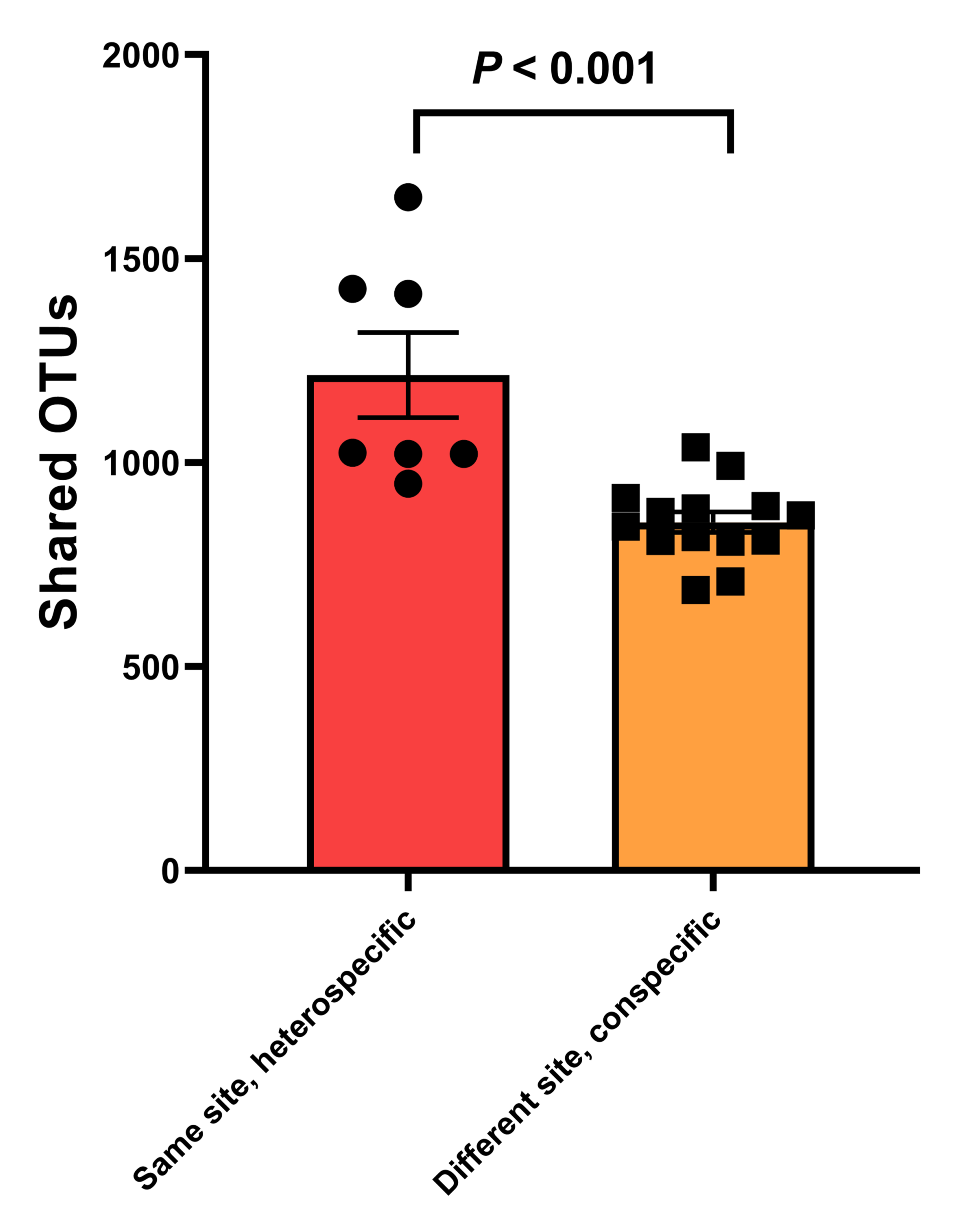
**

**Figure S2 |** Inter-group Wilcoxon test for the number of shared ASVs within the sympatric populations and between allopatric populations.
